# An evaluation of anticoagulation initiation post cardiac surgery in a tertiary cardiac centre

**DOI:** 10.1186/1749-8090-10-S1-A145

**Published:** 2015-12-16

**Authors:** Mohamed A Gulamhussein, Davide Patrini, Jonathan Pararajasingham, Sadeer Fhadil, Paul Wright, Neil Roberts, Nikolaos Panagiotopoulos

**Affiliations:** 1 Cardiothoracic Surgery Department, University College London Hospitals, The Heart Hospital, London, W1G 8PH, UK

## Background/Introduction

Oral anticoagulation with a vitamin K antagonist (usually warfarin) is recommended for lifelong management post mechanical valve insertion. Effects of warfarin are highly variable and dosing outcomes are usually seen after three or four days. This audit was conducted in line with recommendations from patient safety alert 18 to review current practice of warfarin prescribing.

## Aims/Objectives

To assess warfarin initiation in patients post mechanical valve insertion at the Heart Hospital.

## Objectives

• 100% of patients to have a baseline international normalised ratio (INR)

• 100% of patients to have INRs on days 3, 4 and 7 (if appropriate)

• 100% of patients to have dose adjusted on day 4 according to Trust guidelines

• 100% of patients to have consistent dosing on days 1, 2 and 3

• 100% of patients on warfarin prior to admission would have warfarin restarted at the same dose

• 0% of patients to have an INR > 3.5

## Method

This audit was conducted at The Heart Hospital and data collected retrospectively between November 2013 and August 2014. Patient notes, drug charts, electronic CDR and ICIP systems were used to collect inpatient doses, INRs and indications for warfarin. Pre-operative use of warfarin, and dosing, was also determined.

## Results

There were 97 patients identified who had cardiac surgery which included at least one mechanical valve, of which a significant proportion were on warfarin prior to admission (22%, n = 21).

## Results

1. Baseline INR - 100% (97/97)

2. INR Day 2 - 82% (80/97)

3. INR Day 3 - 91% (87/96)

4. INR Day 4 - 87% (79/91)

5. INR Day 7 - 87% (34/39)

6. Consistency of first 3 doses - 30% (29/97)

7. INR > 3.5 - 12% (12/97), > 4 - 6% (6/97)

## Discussion/Conclusion

The effect of warfarin is highly variable and exhibits much inter-patient variability which requires an individually tailored dosing regime when initiating warfarin. This audit suggests that warfarin prescribing is unpredictable following mechanical valve insertion and has led to clinically significant INR excursions of >4.0 in 6% of patients, which in turn suggests that improvements in prescribing could be made.

